# Pain-Specific Benefits of Higher-Dose Neubotulinum Toxin Type A in Cervical Dystonia: A Multicenter Randomized Double-Blind Crossover Trial

**DOI:** 10.3390/toxins18090370

**Published:** 2026-08-28

**Authors:** Arkhom Arayawichanont, Subsai Kongsaengdao

**Affiliations:** 1 Division of Neurology, Department of Medicine, Sunpasitthiprasong Hospital, Ubon Ratchathani 34000, Thailand; 2 Office of Senior Advisor, Department of Medical Services, Ministry of Public Health, Nonthaburi 11000, Thailand; 3 Division of Neurology, Rajavithi Hospital, Department of Medical Services, Ministry of Public Health, Bangkok 10400, Thailand; 4 Division of Neurology, Department of Medicine, College of Medicine, Rangsit University, Bangkok 10400, Thailand

**Keywords:** cervical dystonia, neubotulinum toxin type A, botulinum toxin, pain, randomized crossover trial

## Abstract

Pain is a major contributor to disability in cervical dystonia (CD) and may persist despite improvement in abnormal head posture after botulinum toxin treatment. Whether increasing the dose of Neubotulinum toxin type A (Neu-BoNT-A) provides additional analgesic benefit without compromising safety remains uncertain. In this prospective, multicenter, randomized, double-blind, two-period crossover trial, 50 adults with CD were randomized 1:1 to Neu-BoNT-A 100 U followed by 50 U or 50 U followed by 100 U, with each treatment period lasting 12 weeks. The principal between-dose TWSTRS analysis used a crossover mixed-effects model including treatment, period, and randomized sequence, with participant as a random effect. TWSTRS total, severity, and disability did not differ significantly between regimens. Neu-BoNT-A 100 U produced greater improvement in TWSTRS pain (adjusted 100 U minus 50 U difference, −1.83 points; 95% CI, −2.93 to −0.73; *p* = 0.0011). The pain finding remained significant in a robust GEE sensitivity analysis (difference, −1.83 points; 95% CI, −3.04 to −0.62; *p* = 0.003). CDIP-58 psychosocial functioning also improved more with 100 U (nominal *p* = 0.001). In a Holm sensitivity analysis across all 24 efficacy comparisons, TWSTRS pain and CDIP-58 psychosocial functioning remained significant (adjusted *p* ≈ 0.025 and 0.024, respectively); no other comparison remained significant. No serious adverse events occurred. Neu-BoNT-A 100 U did not improve overall CD severity compared with 50 U but was associated with greater pain relief and improved psychosocial functioning without compromising tolerability. These secondary findings remained significant after multiplicity adjustment but should be interpreted in the context of the nonsignificant overall TWSTRS result and require confirmation.

## 1. Introduction

Cervical dystonia (CD) is the most common focal dystonia in adults and is characterized by involuntary contractions of the cervical muscles that lead to abnormal head posture, impaired function, and substantial reductions in quality of life. Beyond its motor manifestations, CD is increasingly recognized as a multidimensional disorder in which pain, sleep disturbance, fatigue, anxiety, and depression contribute significantly to overall disease burden. Among these non-motor features, pain is particularly prominent, affecting most patients during the course of the disease and frequently representing the symptom that patients identify as most disabling. Consequently, contemporary management of CD has shifted toward improving patient-centered outcomes rather than focusing exclusively on correction of abnormal posture and motor severity [1].

Botulinum toxin type A (BoNT-A) remains the standard first-line treatment for cervical dystonia and is supported by high-quality evidence demonstrating significant improvements in motor severity, disability, and pain. Despite its established efficacy, treatment response is often incomplete. While abnormal head posture frequently improves after injection, residual pain remains a common therapeutic challenge, suggesting that motor improvement and pain relief may not necessarily occur in parallel. These observations have highlighted the need to individualize treatment according to the predominant clinical problems experienced by each patient rather than relying solely on global motor outcome measures [2].

The importance of pain is further supported by real-world observational data. The Cervical Dystonia Patient Registry for Observation of OnabotulinumtoxinA Efficacy (CD PROBE) demonstrated that pain is closely associated with poorer disease-specific quality of life, reduced treatment satisfaction, greater disability, and impaired psychosocial functioning. These findings indicate that effective management of cervical dystonia should address patient-reported outcomes in addition to clinician-rated assessments of motor severity [3].

Recent treatment strategies have therefore emphasized individualized optimization of botulinum toxin therapy, integrating treatment goals that reflect patients’ functional limitations, symptom priorities, and quality of life. Multidisciplinary management, together with personalized adjustment of injection patterns and dosing, has been proposed as an important approach to maximize clinical benefit beyond motor improvement alone [4].

Neubotulinum toxin type A (Neu-BoNT-A) is approved in Thailand for the treatment of cervical dystonia at a total dose of 50 U and has previously demonstrated efficacy and safety comparable with abobotulinumtoxinA in a randomized crossover trial [5]. However, whether increasing the Neu-BoNT-A dose provides additional clinical benefit, particularly with respect to pain, has not been evaluated in a randomized controlled study. Addressing this question is clinically relevant because pain frequently remains the principal residual burden despite otherwise satisfactory motor improvement and may represent an important target for dose optimization.

Therefore, we conducted a prospective, multicenter, randomized, double-blind crossover trial comparing Neu-BoNT-A 50 U and 100 U regimens in adults with cervical dystonia. We hypothesized that increasing the total dose would preferentially improve pain while maintaining an acceptable safety profile, without necessarily producing additional improvement in overall motor severity. Secondary objectives were to compare disease-specific quality of life, general health-related quality of life, depressive symptoms, and treatment safety between the two dosing regimens.

## 2. Results

### 2.1. Participant Characteristics

Between September 2020 and April 2021, 50 adults with cervical dystonia completed both treatment periods of this multicenter randomized crossover trial. The study population comprised 36 women (72%) and 14 men (28%), with a mean age of 52.4 ± 14.2 years. The mean age at diagnosis was 49.0 ± 13.6 years, the mean disease duration was 4.0 ± 3.1 years (range, 1–15 years), and participants had received botulinum toxin treatment for a mean of 3.8 ± 2.7 years before enrollment. All participants had previously undergone regular botulinum toxin injections at approximately 3-month intervals. Although all 50 randomized participants completed both treatment periods, evaluable Week-12 change scores were available for 45 participants for at least one TWSTRS outcome; no missing TWSTRS values were imputed.

Laterocollis was the predominant clinical phenotype (66%), followed by mixed cervical dystonia (20%), anterocollis (8%), and retrocollis (6%). No clinically important coexisting medical conditions were considered likely to interfere with study assessments. The distribution of injected muscles and Neu-BoNT-A doses is summarized in Table 1.

### 2.2. TWSTRS Outcomes

Changes from the pretreatment baseline to Week 12 in TWSTRS outcomes were analyzed using the final crossover mixed-effects model including treatment, period, and randomized treatment sequence, with participant as a random effect. The randomized cohort comprised 50 participants, with 25 assigned to each sequence. The adjusted 100 U minus 50 U difference was not significant for TWSTRS total score (−1.76 points; 95% CI, −5.99 to 2.47; *p* = 0.414), severity (+0.58 points; 95% CI, −3.05 to 4.21; *p* = 0.753), or disability (−0.49 points; 95% CI, −2.12 to 1.14; *p* = 0.554). Neither period nor randomized-sequence effects were statistically significant for these outcomes.

**Figure 1:**
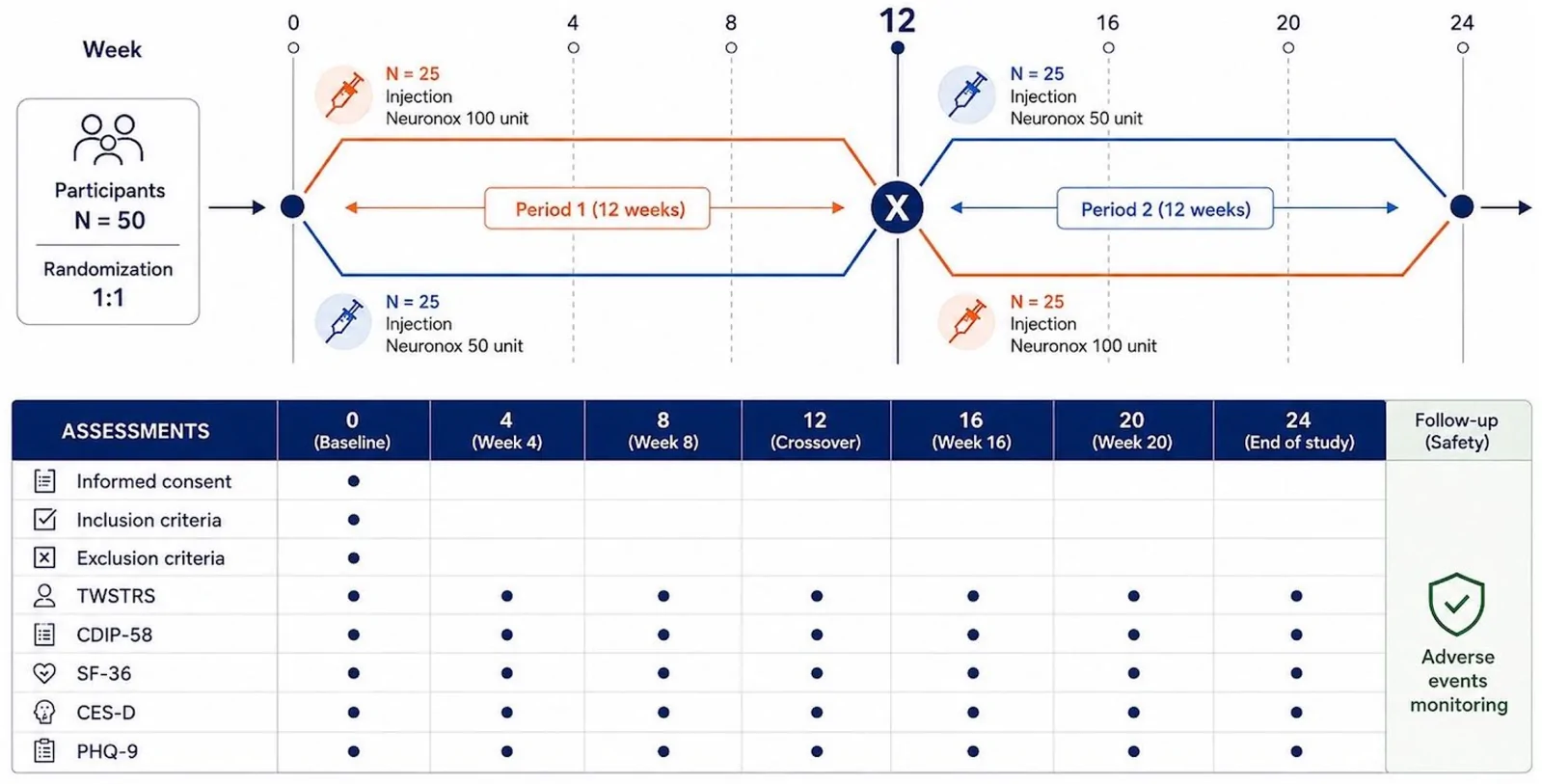
Study design. Fifty participants with cervical dystonia were randomized in a 1:1 ratio to receive Neubotulinum toxin type A (Neu-BoNT-A) 100 U followed by 50 U or 50 U followed by 100 U. Orange and blue lines represent the two randomized treatment sequences, and arrows indicate the direction of treatment progression through the two crossover periods. Each treatment period lasted 12 weeks, with crossover to the alternate regimen at Week 12. Clinical assessments were performed at Weeks 4, 8, and 12 after each treatment. The efficacy analysis presented in this report focuses on the prespecified baseline-to-Week 12 comparison for each treatment period. Assessments included the Toronto Western Spasmodic Torticollis Rating Scale (TWSTRS), Cervical Dystonia Impact Profile-58 (CDIP-58), 36-Item Short Form Health Survey (SF-36), Center for Epidemiologic Studies Depression Scale (CES-D), and Patient Health Questionnaire-9 (PHQ-9). Adverse events were monitored throughout the 24-week study.

In contrast, the TWSTRS pain subscore demonstrated a significant adjusted between-regimen difference. In the crossover model, the adjusted 100 U minus 50 U difference was −1.83 points (95% CI, −2.93 to −0.73; *p* = 0.0011), favoring 100 U. Because the participant random-intercept variance was estimated at the boundary, a Gaussian GEE sensitivity analysis with robust standard errors and an unstructured within-participant correlation was performed; the pain result remained significant (difference, −1.83 points; 95% CI, −3.04 to −0.62; *p* = 0.003). A first-period-only sensitivity analysis showed a concordant direction (−1.50 points; 95% CI, −3.18 to 0.17; *p* = 0.077) but lower precision. The study design and assessment schedule are summarized in Figure 1.

### 2.3. Disease-Specific Quality of Life

Overall CDIP-58 scores did not differ significantly between the two treatment regimens (*p* = 0.45). Similarly, no significant between-regimen differences were identified in the head and neck symptoms (*p* = 0.53), pain and discomfort (*p* = 0.65), upper-limb activities (*p* = 0.68), walking (*p* = 0.55), annoyance (*p* = 0.38), or mood (*p* = 0.11) domains.

**Figure 2:**
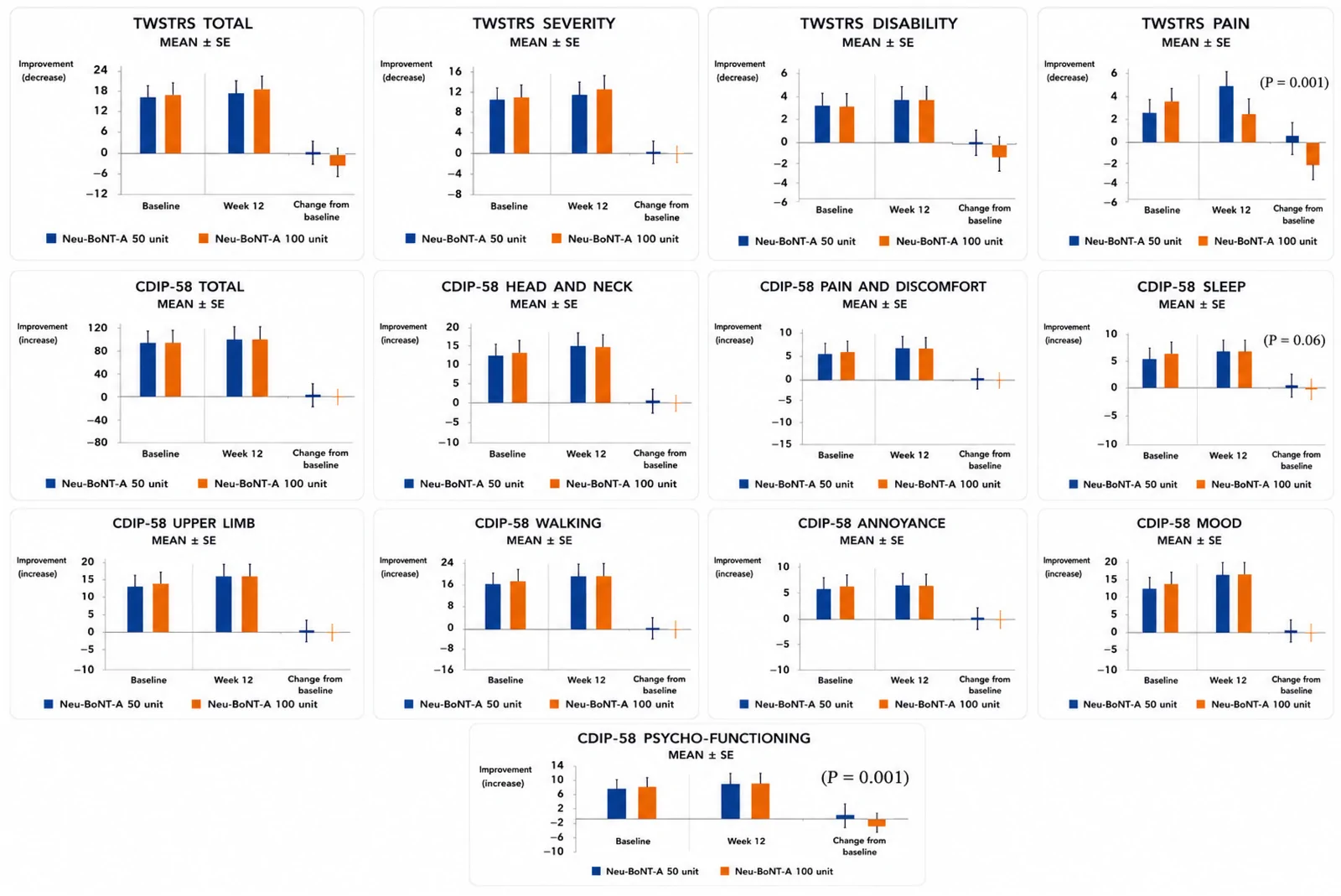
Changes in clinical severity and disease-specific quality of life following treatment with Neubotulinum toxin type A (Neu-BoNT-A) 50 U and 100 U. Bar charts present mean scores with standard errors (SE) at the pretreatment baseline and Week 12, together with the mean change from baseline, for the Toronto Western Spasmodic Torticollis Rating Scale (TWSTRS) total score and its severity, disability, and pain subscores and for the Cervical Dystonia Impact Profile-58 (CDIP-58) total and domain scores. Blue bars represent the Neu-BoNT-A 50 U regimen, and orange bars represent the 100 U regimen. Values are mean ± SE, and error bars indicate SE. Lower scores indicate improvement for both TWSTRS and CDIP-58 outcomes. *p*-values are displayed for selected between-regimen comparisons. Abbreviations: CDIP-58, Cervical Dystonia Impact Profile-58; Neu-BoNT-A, Neubotulinum toxin type A; SE, standard error; TWSTRS, Toronto Western Spasmodic Torticollis Rating Scale; U, units.

The sleep domain showed a trend toward greater improvement with the 100 U regimen, although the nominal difference did not reach statistical significance (*p* = 0.06). In contrast, psychosocial functioning improved significantly more following the 100 U regimen than after the 50 U regimen (mean change, −1.6 ± 0.7 vs. +2.2 ± 0.8; nominal *p* = 0.001), with lower CDIP-58 scores indicating better disease-specific quality of life (Table 2 and Figure 2). In the Holm sensitivity analysis across all 24 efficacy comparisons, CDIP-58 psychosocial functioning remained statistically significant (Holm-adjusted *p* = 0.024).

### 2.4. General Health-Related Quality of Life

**Figure 3:**
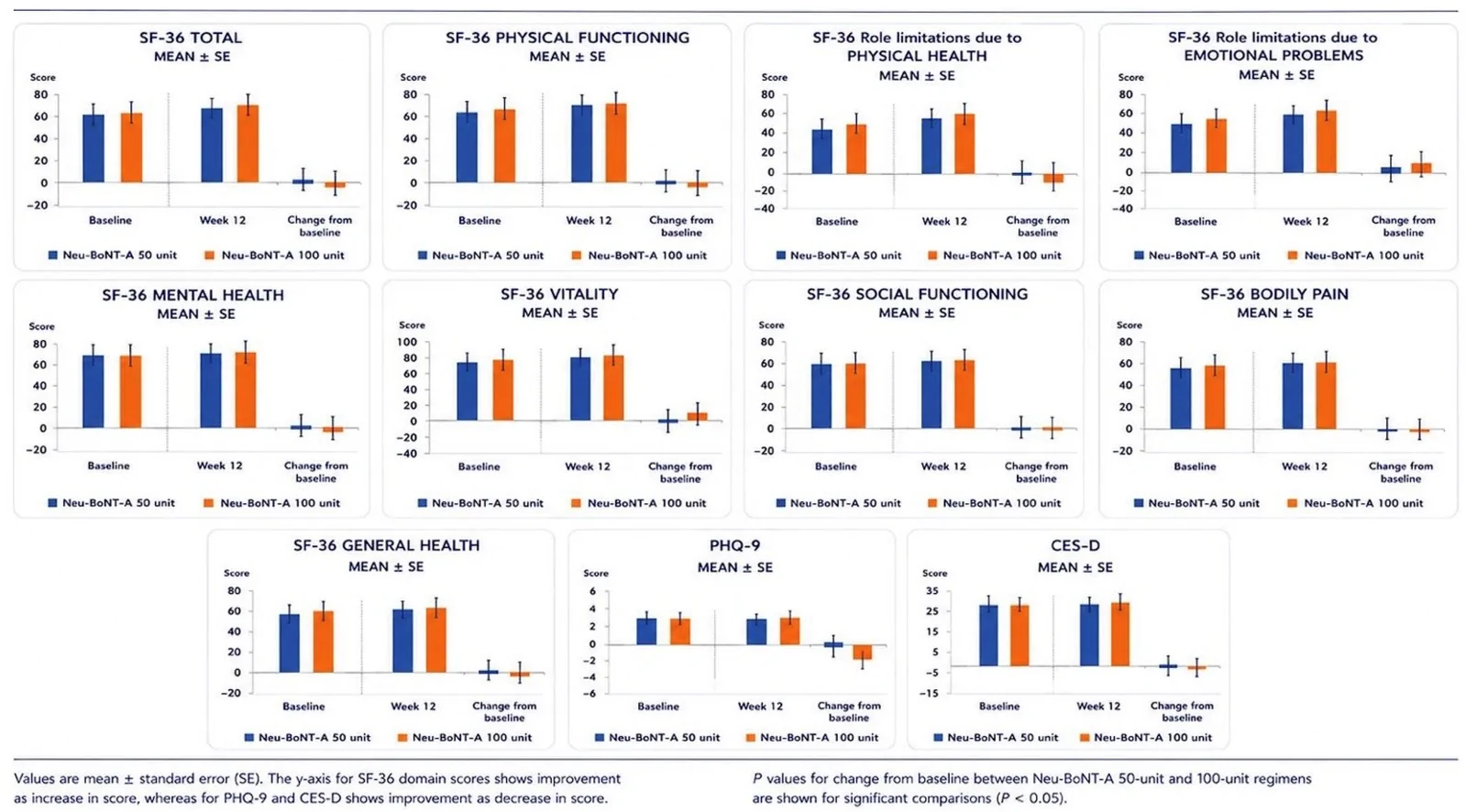
Changes in health-related quality of life and depressive symptoms following treatment with Neubotulinum toxin type A (Neu-BoNT-A) 50 U and 100 U. Bar charts present mean scores with standard errors (SEs) at the pretreatment baseline and Week 12, together with the mean change from baseline, for the 36-Item Short Form Health Survey (SF-36) total score and its domains, including physical functioning, role limitations due to physical health, role limitations due to emotional problems, mental health, vitality, social functioning, bodily pain, and general health. Depressive symptoms were assessed using the Patient Health Questionnaire-9 (PHQ-9) and the Center for Epidemiologic Studies Depression Scale (CES-D). Blue bars represent the Neu-BoNT-A 50 U regimen, and orange bars represent the 100 U regimen. Error bars indicate SE. Higher SF-36 scores indicate better health-related quality of life, whereas lower PHQ-9 and CES-D scores indicate fewer depressive symptoms. Abbreviations: CES-D, Center for Epidemiologic Studies Depression Scale; Neu-BoNT-A, Neubotulinum toxin type A; PHQ-9, Patient Health Questionnaire-9; SE, standard error; SF-36, 36-Item Short Form Health Survey; U, units.

No significant between-regimen differences were observed in the SF-36 total score or any individual domain. Physical functioning, role limitations due to physical health, role limitations due to emotional problems, vitality, mental health, social functioning, bodily pain, and general health perception remained comparable between the 50 U and 100 U regimens (all *p* > 0.05) (Table 2 and Figure 3).

**Figure 4:**
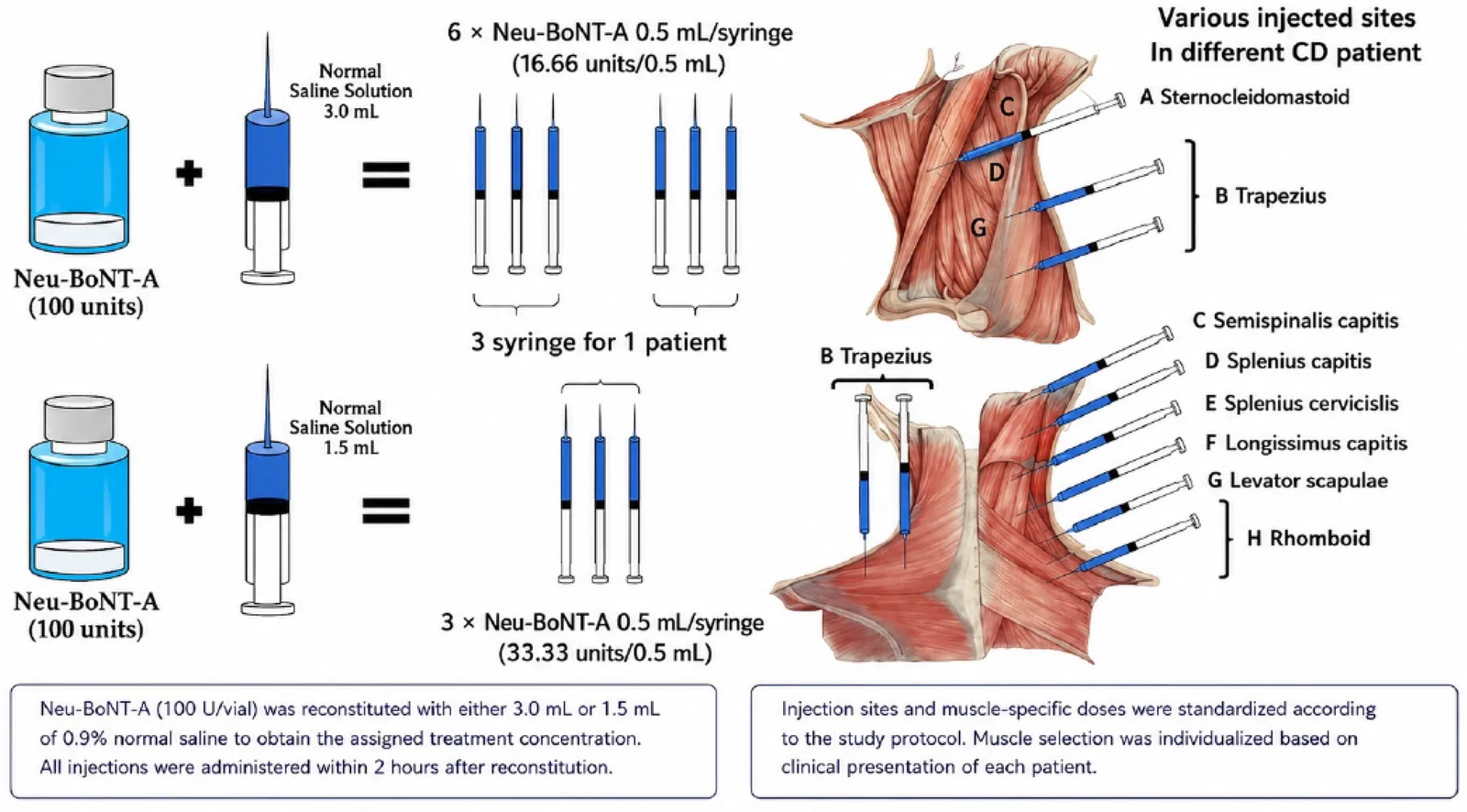
Reconstitution of Neubotulinum toxin type A (Neu-BoNT-A) and individualized injection protocol for cervical dystonia. For the 50 U regimen, a 100 U vial of Neu-BoNT-A was reconstituted with 3.0 mL of preservative-free 0.9% normal saline, producing a concentration of 33.33 U/mL. Three 0.5 mL syringes, each containing 16.67 U, were administered to each participant, resulting in a total dose of 50 U in 1.5 mL. For the 100 U regimen, a 100 U vial was reconstituted with 1.5 mL of saline, producing a concentration of 66.67 U/mL. Three 0.5 mL syringes, each containing 33.33 U, were administered, resulting in a total dose of 100 U in 1.5 mL. For each participant, the selected muscles, anatomical injection sites, number of injection points, and relative dose distribution were determined before randomization and remained unchanged during both treatment periods. Thus, each participant received the same individualized injection pattern and total injection volume in both periods, with only the Neu-BoNT-A concentration and total assigned dose differing. The illustrated sites are representative examples and include the sternocleidomastoid, trapezius, semispinalis capitis, splenius capitis, splenius cervicis, longissimus capitis, levator scapulae, and rhomboid muscles. Abbreviations: CD, cervical dystonia; Neu-BoNT-A, Neubotulinum toxin type A; U, units.

The Neu-BoNT-A reconstitution and individualized injection protocol are illustrated in Figure 4.

### 2.5. Depressive Symptoms

Changes in depressive symptoms were similar between treatment regimens. Neither CES-D (*p* = 0.19) nor PHQ-9 (*p* = 0.17) demonstrated significant between-regimen differences, indicating no detectable dose-dependent effect of Neu-BoNT-A on depressive symptom severity during the study period (Table 2 and Figure 3).

### 2.6. Safety

Both Neu-BoNT-A regimens were well tolerated. No common treatment-related adverse effects or serious adverse events were reported during either treatment period.

## 3. Discussion

The present multicenter, randomized, double-blind crossover trial found that increasing the Neu-BoNT-A dose from 50 U to 100 U did not produce additional improvement in overall cervical dystonia severity or disability. However, the higher dose was associated with greater improvement in the TWSTRS pain subscore and with better psychosocial functioning on the CDIP-58. In the final crossover model accounting for treatment period and randomized sequence, the adjusted pain difference was −1.83 points (95% CI, −2.93 to −0.73; *p* = 0.0011), and the finding remained significant in a robust GEE sensitivity analysis. TWSTRS pain and CDIP-58 psychosocial functioning remained significant after Holm adjustment across all 24 efficacy comparisons (adjusted *p* ≈ 0.025 and 0.024, respectively). No treatment-related serious adverse events were observed. These findings remain secondary and should be interpreted in the context of the nonsignificant overall TWSTRS comparison.

Our findings extend previous evidence regarding Neu-BoNT-A in cervical dystonia. In our earlier multicenter crossover trial, Neu-BoNT-A 50 U demonstrated efficacy and safety comparable with abobotulinumtoxinA 250 U, establishing the approved 50 U regimen as an effective therapeutic option [5]. The current study addressed a different and clinically relevant question by comparing two doses of the same formulation. The observed pattern suggests that motor benefit may plateau at the lower dose in previously treated patients, whereas pain may remain amenable to further dose optimization.

The selective analgesic signal observed in this study is consistent with the growing recognition that pain represents a distinct therapeutic target in cervical dystonia. Contemporary reviews and systematic evidence indicate that although BoNT-A effectively improves motor symptoms, treatment goals should extend beyond correction of abnormal posture to include pain control, functional recovery, and patient-reported outcomes. Pain is frequently one of the most disabling manifestations of cervical dystonia and contributes substantially to treatment satisfaction and quality of life [1,2,3,6].

Greater pain reduction was accompanied by improved psychosocial functioning despite the absence of significant differences in TWSTRS total score or generic health-related quality of life. Although causality cannot be established, this pattern suggests that improvements in pain may translate into meaningful benefits in social participation and psychosocial well-being even when overall motor severity changes little. Previous studies have likewise shown that disease-specific quality-of-life instruments may be more sensitive than generic measures for detecting clinically relevant treatment effects in cervical dystonia [7].

By contrast, no significant between-dose differences were observed in SF-36, PHQ-9, or CES-D outcomes. Generic health-related quality-of-life instruments may lack sensitivity to detect focused treatment effects, whereas psychological symptoms in cervical dystonia are influenced by multiple biological and psychosocial factors that extend beyond motor dysfunction or pain alone. Consequently, optimization of botulinum toxin therapy should not be expected to fully address depression or broader psychological distress, for which multidisciplinary management may remain appropriate [4,8,9].

Both Neu-BoNT-A regimens demonstrated excellent tolerability, with no common treatment-related adverse effects or serious adverse events observed. Although the study was not powered to evaluate rare adverse events or formally establish safety equivalence, these findings are reassuring and support the feasibility of individualized dose escalation when clinically indicated. Nevertheless, careful muscle selection and dose distribution remain essential to minimize dose-related complications, particularly dysphagia and neck weakness, which are recognized adverse effects of botulinum toxin therapy [2,6].

This study has several important strengths. The randomized double-blind crossover design enabled direct within-participant comparison while minimizing interindividual variability. Additional strengths include multicenter recruitment, standardized injection procedures, identical total injection volumes to preserve masking, and the combined use of clinician-rated and patient-reported outcome measures.

Several limitations should be acknowledged. First, the relatively small sample size limited statistical power for secondary outcomes and uncommon adverse events. Second, although outcomes were assessed at Weeks 4, 8, and 12 after each injection, the present report focuses on the protocol-defined overall treatment comparison at Week 12; therefore, these findings primarily reflect response at the end of each treatment cycle rather than the earlier peak treatment effect. Third, although the two positive secondary findings remained significant in a Holm sensitivity analysis across all 24 efficacy comparisons, pain and psychosocial functioning were secondary outcomes, and the trial was not prospectively powered for these endpoints. Fourth, the final crossover analysis found no statistically significant period or randomized-sequence effect, but lack of a sequence effect does not prove absence of biological carryover; residual carryover cannot be completely excluded. The participant random-intercept variance was estimated at the boundary, although the pain finding remained significant in a robust GEE sensitivity analysis. Finally, all participants had previous exposure to botulinum toxin, which may limit generalizability to treatment-naive patients and may have contributed to a treatment-response plateau for overall motor outcomes.

Overall, the present findings support a patient-centered approach to Neu-BoNT-A treatment in cervical dystonia. Rather than pursuing routine dose escalation to improve motor outcomes, clinicians may consider increasing the dose in patients whose principal residual burden is persistent pain despite otherwise satisfactory motor control. The pain signal was consistent across the crossover mixed-effects and robust GEE analyses and remained significant after Holm multiplicity adjustment; nevertheless, pain was a secondary endpoint, and a validated TWSTRS-pain-specific MCID was not available for this comparison. Larger randomized trials with pain designated as a prespecified primary endpoint and longitudinal evaluation throughout the injection cycle are warranted to confirm these findings and refine individualized dosing strategies.

## 4. Conclusions

In this multicenter, randomized, double-blind crossover trial, increasing the Neu-BoNT-A dose from 50 U to 100 U did not improve overall cervical dystonia severity but was associated with greater pain relief and improved disease-specific psychosocial functioning without an observed increase in serious adverse events. The pain difference remained significant in the final treatment-period-sequence crossover model and in a robust GEE sensitivity analysis, and both TWSTRS pain and CDIP-58 psychosocial functioning remained significant after Holm adjustment across all 24 efficacy comparisons (adjusted *p* ≈ 0.025 and 0.024, respectively). Rather than supporting routine use of higher doses, these results favor an individualized, symptom-oriented treatment strategy. Because pain and psychosocial functioning were secondary outcomes and the clinical importance of the approximately 1.8-point adjusted pain difference has not been established against a validated pain-specific MCID, confirmation in larger randomized studies is warranted.

## 5. Materials and Methods

### 5.1. Study Design

This investigator-initiated study was a prospective, multicenter, randomized, double-blind, two-period crossover trial comparing two total doses of Neu-BoNT-A for the treatment of CD. Participants were recruited from four tertiary referral hospitals in Thailand: Rajavithi Hospital, Sunpasitthiprasong Hospital, Lampang Hospital, and Suratthani Hospital.

**Figure 5:**
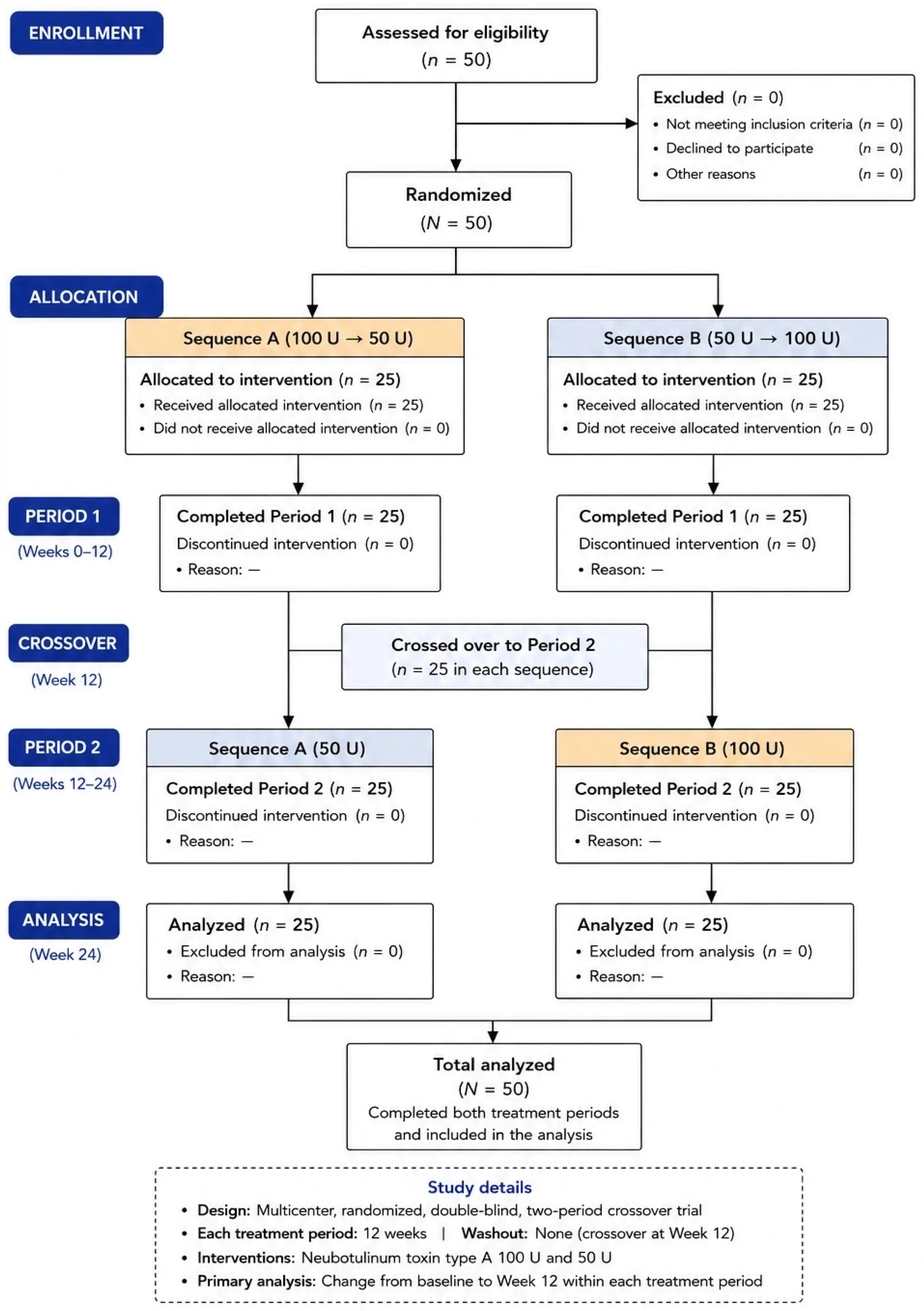
CONSORT flow diagram. Participant flow through the multicenter, randomized, double-blind, two-period crossover trial comparing Neubotulinum toxin type A 50 U and 100 U in adults with cervical dystonia. Fifty participants were randomized 1:1 to receive 100 U followed by 50 U or 50 U followed by 100 U. All 50 participants completed both 12-week treatment periods, crossed over at Week 12, and were included in the analysis.

Participants were randomly assigned to receive either Neu-BoNT-A 50 U followed by 100 U or the reverse treatment sequence. Each treatment period lasted 12 weeks, after which participants crossed over to the alternate regimen. The crossover design enabled direct within-participant comparison while minimizing interindividual variability (Figure 5).

The study complied with the Declaration of Helsinki and the International Council for Harmonisation Guideline for Good Clinical Practice. The protocol was approved by the Ministry of Public Health Ethics Committee (Approval No. 33/2562; EC-MOPH No. 33/2019), and all participants provided written informed consent before enrollment. The study was conducted as an extension of the registered ClinicalTrials.gov trial (NCT03805152).

### 5.2. Participants

Adults aged 18 years or older with clinically diagnosed cervical dystonia who were able to communicate in Thai and complete all study assessments were eligible. Women of childbearing potential were required to have a negative pregnancy test before enrollment and to use effective contraception throughout study participation.

Key exclusion criteria included pregnancy or breastfeeding; hypersensitivity to botulinum toxin; neuromuscular junction disorders, including myasthenia gravis and Lambert–Eaton syndrome; clinically significant coagulation disorders; severe cardiovascular disease; inflammatory cervical arthritis; concomitant aminoglycoside or curare-like medications; participation in another interventional study; planned elective surgery during follow-up; or any medical or psychiatric condition considered by the investigators likely to interfere with treatment administration or outcome assessment.

### 5.3. Randomization and Blinding

Participants were randomized in a 1:1 ratio using a computer-generated allocation sequence (Randomizer.org, accessed on 12 June 2019). Treatment assignments were concealed in sequentially numbered opaque sealed envelopes and were accessible only to the pharmacist responsible for study-drug preparation.

The trial was conducted under double-blind conditions. Participants, treating physicians, investigators, outcome assessors, and statisticians remained unaware of treatment allocation throughout the study. To preserve masking, the total injection volume administered to each participant was identical for both treatment regimens.

### 5.4. Intervention

Neu-BoNT-A was supplied in lyophilized 100 U vials. For the 50 U regimen, each vial was reconstituted with 3.0 mL of preservative-free 0.9% normal saline, producing a concentration of 33.33 U/mL. Three 0.5 mL syringes, each containing 16.67 U, were administered to each participant, resulting in a total dose of 50 U in 1.5 mL. For the 100 U regimen, each vial was reconstituted with 1.5 mL of saline, producing a concentration of 66.67 U/mL. Three 0.5 mL syringes, each containing 33.33 U, were administered, resulting in a total dose of 100 U in 1.5 mL. Thus, the total injection volume was identical for both regimens. All injections were administered within two hours after reconstitution.

For each participant, target muscles were selected using clinical examination, anatomical landmarks, and the participant’s established individual treatment-response pattern from previous botulinum toxin injections; electromyography and ultrasound were not routinely used. All participants were experienced with botulinum toxin treatment before enrollment, having received regular injections for a mean of approximately 3.8 years. The selected muscles, anatomical injection sites, number of injection points, and relative dose distribution were determined before randomization and remained unchanged throughout both treatment periods. Thus, each participant received the same individualized injection pattern in both periods, with only the toxin concentration and assigned total dose differing. Target muscles could include the sternocleidomastoid, trapezius, splenius capitis, splenius cervicis, levator scapulae, semispinalis capitis, longissimus capitis, rhomboid, and other clinically involved cervical muscles when indicated.

### 5.5. Follow-Up

Participants underwent standardized clinical evaluations immediately before each treatment and at Weeks 4, 8, and 12 after each injection. After completion of the first 12-week treatment period, participants crossed over to the alternate regimen and underwent the same assessment schedule during the second treatment period. In accordance with the study protocol, the present analysis focuses on the overall treatment response from the pretreatment baseline to Week 12 of each treatment period. Safety was monitored continuously throughout the 24-week study.

### 5.6. Outcome Measures

The prespecified overall efficacy evaluation reported in this article was the change from the pretreatment baseline to Week 12 of each treatment period in the Toronto Western Spasmodic Torticollis Rating Scale (TWSTRS), including the total score and the severity, disability, and pain subscores. Although assessments were conducted at Weeks 4, 8, and 12 after each injection, the Week 12 assessment was used for the protocol-defined overall treatment comparison. To improve assessment consistency, TWSTRS evaluations were performed using a standardized video-recording protocol.

Secondary outcomes included disease-specific quality of life assessed using the validated Thai version of the Cervical Dystonia Impact Profile-58 (CDIP-58), generic health-related quality of life measured using the Thai version of the 36-Item Short Form Health Survey (SF-36), depressive symptoms evaluated using the Patient Health Questionnaire-9 (PHQ-9) and Center for Epidemiologic Studies Depression Scale (CES-D), and treatment safety.

Safety outcomes included treatment-emergent adverse events, serious adverse events, and overall treatment tolerability recorded throughout the study.

### 5.7. Statistical Analysis

Baseline demographic and clinical characteristics are presented using descriptive statistics. Continuous variables are expressed as mean ± standard deviation (SD), whereas treatment outcomes are presented as mean ± standard error (SE). Categorical variables are summarized as frequencies and percentages.

Within-regimen changes from baseline to Week 12 were summarized descriptively. The principal between-dose TWSTRS analysis used a linear mixed-effects crossover model with treatment (100 U versus 50 U), treatment period, and randomized treatment sequence as fixed effects and participant as a random effect. The randomized cohort comprised 50 participants, with 25 assigned to each sequence. Change was defined as Week 12 minus pretreatment baseline; therefore, negative treatment differences favored 100 U. Model-estimated treatment differences, 95% confidence intervals, and two-sided *p*-values are reported. Because the fitted participant random-intercept variance was estimated at the boundary, a Gaussian generalized estimating equation (GEE) model with robust standard errors and an unstructured within-participant correlation was used as a sensitivity analysis. A first-period-only analysis was additionally examined as a carryover-oriented sensitivity analysis. Statistical significance was defined as a two-sided *p* < 0.05.

Sample size was determined according to the anticipated difference in the TWSTRS total score between treatment regimens, assuming 80% statistical power and a two-sided significance level of 0.05.

Because multiple secondary outcomes and questionnaire domains were evaluated, a Holm step-down multiplicity sensitivity analysis was applied across all 24 efficacy comparisons as a single family. For the four TWSTRS outcomes, the updated crossover-model *p*-values were used; for the remaining efficacy outcomes, the nominal *p*-values reported in Table 2 were retained. Nominal and Holm-adjusted *p*-values are reported transparently. Secondary outcomes remained exploratory even when statistically significant after adjustment and were interpreted in the context of the nonsignificant overall TWSTRS comparison.

Original descriptive and within-regimen analyses were performed using StatXact version 6.2 (Cytel Studio^®^, Cytel Inc., Cambridge, MA, USA). The revision crossover mixed-effects, GEE, first-period sensitivity, and multiplicity analyses were performed in Python 3.13 using statsmodels 0.14.6 and SciPy 1.17.0. Additional details of the crossover mixed-effects, GEE, first-period sensitivity, and multiplicity analyses are provided in the Supplementary Materials.

## Figures and Tables

**Table 1:** Distribution of Neubotulinum toxin type A (Neu-BoNT-A) doses administered to individual muscles during treatment with the 100 U and 50 U regimens. Values are presented as the mean, standard deviation (SD), minimum (Min), maximum (Max), and range of the injected dose per muscle. The final column indicates the number of muscle-treatment observations for each muscle across both treatment periods. Muscle selection and dose allocation were individualized according to each participant’s clinical presentation and were fixed for that participant before randomization. Abbreviations: Neu-BoNT-A, Neubotulinum toxin type A; SD, standard deviation; U, units.

Dosage (Unit Per Muscle)	Neu-BoNT-A 100 Unit					Neu-BoNT-A 50 Unit					Number of Injected Muscle
	Mean	SD	Min	Max	Range	Mean	SD	Min	Max	Range	
**Trapezius**	24.7	13.4	8.3	66.7	58.3	12.4	6.7	4.2	33.3	29.2	86
**Sternocleidomastoid**	17.3	8.5	3.3	33.3	30.0	8.7	4.3	1.7	16.7	15.0	60
**Splenius capitis**	20.0	6.7	8.3	33.3	25.0	10.0	3.3	4.2	16.7	12.5	51
**Splenius cervicalis**	13.4	8.4	4.2	33.3	29.2	6.7	4.2	2.1	16.7	14.6	21
**Levator scapulae**	16.7	3.4	8.3	25.0	16.7	7.4	2.5	0.0	8.3	8.3	13
**Semispinalis capitis**	16.1	13.4	6.7	33.3	26.7	9.4	6.6	3.3	16.7	13.3	6
**Longissimus capitis**	9.7	1.9	8.3	11.0	2.7	4.8	0.9	4.2	5.5	1.3	2
**Rhomboideus**	16.7	0.0	16.7	16.7	0.0	8.3	0.0	8.3	8.3	0.0	1

**Table 2:** Changes in clinical severity, disease-specific quality of life, health-related quality of life, and depressive symptoms following treatment with Neubotulinum toxin type A (Neu-BoNT-A) at 50 U and 100 U. Values are presented as mean (standard error [SE]) at the pretreatment baseline and Week 12 of each treatment period, together with the mean change from baseline. For TWSTRS outcomes, the principal between-dose inference is based on the final crossover mixed-effects model including treatment, period, and randomized sequence, with participant as a random effect; the corresponding model estimates and *p*-values are reported in the Results. Secondary-outcome comparisons are shown as nominal *p*-values. Holm’s step-down procedure was applied across all 24 efficacy comparisons; TWSTRS pain and CDIP-58 psychosocial functioning remained significant after adjustment (Holm-adjusted *p* ≈ 0.025 and 0.024, respectively), and no other comparison remained significant. Abbreviations: CDIP-58, Cervical Dystonia Impact Profile-58; CES-D, Center for Epidemiologic Studies Depression Scale; Neu-BoNT-A, Neubotulinum toxin type A; PHQ-9, Patient Health Questionnaire-9; SE, standard error; SF-36, 36-Item Short Form Health Survey; TWSTRS, Toronto Western Spasmodic Torticollis Rating Scale; U, units.

Questionnaires	Treatment Groups	Score at Baseline Mean (SE)	Score at Week 12 Mean (SE)	Mean Change from Baseline	*p*-Value After Treatment
**Primary Outcomes**					
TWSTRS total	Neu-BoNT-A 50 unit	17.5 (1.8)	17.7 (2.2)	0.2 (1.3)	0.94
	Neu-BoNT-A 100 unit	18.3 (2.0)	18.6 (1.7)	−1.6 (1.5)	0.92
	***p*** **-value between treatment groups**	**0.77**	**0.76**	**0.36**	
TWSTRS severity	Neu-BoNT-A 50 unit	11.3 (1.3)	11.0 (1.5)	−0.3 (1.2)	0.87
	Neu-BoNT-A 100 unit	11.6 (1.3)	12.6 (1.3)	0.1 (1.2)	0.61
	***p*** **-value between treatment groups**	**0.86**	**0.43**	**0.78**	
TWSTRS disability	Neu-BoNT-A 50 unit	3.1 (0.8)	2.9 (0.8)	0.06 (0.4)	0.82
	Neu-BoNT-A 100 unit	2.9 (0.8)	2.7 (0.7)	−0.4 (0.6)	0.92
	***p*** **-value between treatment groups**	**0.81**	**0.91**	**0.51**	
TWSTRS pain	Neu-BoNT-A 50 unit	3.0 (0.5)	3.6 (0.5)	0.6 (0.4)	0.29
	Neu-BoNT-A 100 unit	3.8 (0.5)	2.7 (0.4)	−1.2 (0.3)	0.17
	***p*** **-value between treatment groups**	**0.32**	**0.22**	**0.001**	
CDIP-58 total	Neu-BoNT-A 50 unit	97.0 (5.1)	100.8 (5.8)	3.0 (4.7)	0.61
	Neu-BoNT-A 100 unit	99.3 (5.8)	97.8 (5.0)	−1.4 (3.5)	0.95
	***p*** **-value between treatment groups**	**0.76**	**0.69**	**0.45**	
CDIP-58 head and neck	Neu-BoNT-A 50 unit	14.1 (0.8)	14.8 (0.9)	0.5 (0.7)	0.57
	Neu-BoNT-A 100 unit	14.4 (0.8)	14.2 (0.8)	−0.1 (0.7)	0.99
	***p*** **-value between treatment groups**	**0.78**	**0.63**	**0.53**	
CDIP-58 pain and discomfort	Neu-BoNT-A 50 unit	12.3 (0.7)	12.1 (0.7)	−0.6 (0.7)	0.80
	Neu-BoNT-A 100 unit	12.5 (0.7)	12.1 (0.7)	−0.1 (0.7)	0.86
	***p*** **-value between treatment groups**	**0.87**	**0.95**	**0.65**	
CDIP-58 sleep	Neu-BoNT-A 50 unit	6.6 (0.6)	7.7 (0.6)	0.9 (0.5)	0.22
	Neu-BoNT-A 100 unit	7.5 (0.5)	6.9 (0.5)	−0.3 (0.3)	0.56
	***p*** **-value between treatment groups**	**0.32**	**0.35**	**0.06**	
CDIP-58 upper limb	Neu-BoNT-A 50 unit	18.3 (1.1)	18.2 (1.2)	−0.3 (1.1)	0.93
	Neu-BoNT-A 100 unit	17.7 (1.1)	18.5 (1.3)	0.3 (0.9)	0.51
	***p*** **-value between treatment groups**	**0.67**	**0.85**	**0.68**	
CDIP-58 walking	Neu-BoNT-A 50 unit	19.3 (1.4)	19.5 (1.3)	0.3 (1.1)	0.89
	Neu-BoNT-A 100 unit	19.3 (1.4)	19.0 (1.3)	−0.6 (1.0)	0.97
	***p*** **-value between treatment groups**	**0.99**	**0.78**	**0.55**	
CDIP-58 annoyance	Neu-BoNT-A 50 unit	15.5 (1.1)	16.5 (1.1)	0.9 (1.0)	0.57
	Neu-BoNT-A 100 unit	16.0 (1.1)	15.4 (1.0)	−0.1 (0.7)	0.77
	***p*** **-value between treatment groups**	**0.79**	**0.52**	**0.38**	
CDIP-58 mood	Neu-BoNT-A 50 unit	10.5 (0.7)	11.8 (0.8)	1.3 (0.6)	0.23
	Neu-BoNT-A 100 unit	11.8 (0.8)	11.3 (0.8)	−0.2 (0.7)	0.76
	***p*** **-value between treatment groups**	**0.24**	**0.70**	**0.11**	
CDIP-58 psychosocial functioning	Neu-BoNT-A 50 unit	12.2 (0.8)	14.0 (1.0)	2.2 (0.8)	0.16
	Neu-BoNT-A 100 unit	14.0 (1.0)	12.2 (0.8)	−1.6 (0.7)	0.15
	***p*** **-value between treatment groups**	**0.16**	**0.16**	**0.001**	
**Secondary outcomes**					
SF-36 total	Neu-BoNT-A 50 unit	59.2 (2.8)	59.9 (3.1)	0.5 (2.7)	0.87
	Neu-BoNT-A 100 unit	64.4 (3.0)	62.8 (3.0)	−2.1 (3.8)	0.70
	***p*** **-value between treatment groups**	**0.21**	**0.51**	**0.56**	
SF-36 physical functioning	Neu-BoNT-A 50 unit	65.8 (3.6)	63.5 (4.7)	−3.8 (5.0)	0.70
	Neu-BoNT-A 100 unit	70.7 (4.0)	67.6 (4.3)	−3.9 (5.1)	0.61
	***p*** **-value between treatment groups**	**0.37**	**0.52**	**0.98**	
SF-36 limitations physical health	Neu-BoNT-A 50 unit	45.4 (6.9)	45.5 (7.9)	−1.6 (8.5)	0.98
	Neu-BoNT-A 100 unit	53.5 (7.4)	55.8 (6.8)	1.6 (10.2)	0.81
	***p*** **-value between treatment groups**	**0.42**	**0.32**	**0.80**	
SF-36 limitation emotion	Neu-BoNT-A 50 unit	47.6 (7.5)	45.1 (8.0)	−3.6 (8.7)	0.82
	Neu-BoNT-A 100 unit	52.0 (7.5)	56.0 (7.5)	4.3 (10.8)	0.70
	***p*** **-value between treatment groups**	**0.68**	**0.32**	**0.56**	
SF-36 mental health	Neu-BoNT-A 50 unit	61.0 (3.2)	64.1 (2.4)	0.2 (3.5)	0.45
	Neu-BoNT-A 100 unit	67.2 (3.0)	64.4 (2.9)	−1.9 (3.8)	0.52
	***p*** **-value between treatment groups**	**0.16**	**0.92**	**0.68**	
SF-36 vitality	Neu-BoNT-A 50 unit	76.8 (2.8)	81.8 (2.7)	1.7 (3.8)	0.20
	Neu-BoNT-A 100 unit	82.2 (3.0)	80.2 (2.6)	−2.5 (3.6)	0.62
	***p*** **-value between treatment groups**	**0.20**	**0.67**	**0.42**	
SF-36 social functioning	Neu-BoNT-A 50 unit	62.3 (1.0)	61.6 (1.0)	−2.0 (1.9)	0.64
	Neu-BoNT-A 100 unit	62.1 (1.2)	62.5 (1.5)	1.0 (1.7)	0.80
	***p*** **-value between treatment groups**	**0.90**	**0.55**	**0.23**	
SF-36 bodily pain	Neu-BoNT-A 50 unit	60.3 (1.5)	60.7 (1.2)	−1.3 (2.1)	0.81
	Neu-BoNT-A 100 unit	57.3 (1.5)	59.5 (1.7)	2.3 (2.1)	0.33
	***p*** **-value between treatment groups**	**0.16**	**0.55**	**0.23**	
SF-36 general health	Neu-BoNT-A 50 unit	52.6 (3.3)	52.4 (2.9)	−2.3 (3.7)	0.87
	Neu-BoNT-A 100 unit	56.4 (3.2)	55.2 (3.4)	−1.3 (3.6)	0.80
	***p*** **-value between treatment groups**	**0.42**	**0.54**	**0.83**	
CES-D	Neu-BoNT-A 50 unit	31.1 (1.4)	31.8 (1.2)	1.5 (1.0)	0.71
	Neu-BoNT-A 100 unit	31.4 (1.3)	30.3 (1.1)	−0.4 (0.9)	0.52
	***p*** **-value between treatment groups**	**0.85**	**0.39**	**0.19**	
PHQ-9	Neu-BoNT-A 50 unit	3.6 (0.7)	4.3 (0.8)	0.4 (0.8)	0.53
	Neu-BoNT-A 100 unit	4.5 (0.7)	3.5 (0.5)	−1.0 (0.7)	0.29
	***p*** **-value between treatment groups**	**0.42**	**0.40**	**0.17**	

## Data Availability

The data presented in this study are available from the corresponding author upon reasonable request. The data are not publicly available because they contain individual-level clinical trial data and are subject to participant privacy and ethical restrictions.

## Supplementary Materials

The following supporting information can be downloaded at https://www.mdpi.com/article/10.3390/toxins18090370/s1, Supplementary File: Statistical Report V3—FINAL.
