# Supplementary material for: Pain-Specific Benefits of Higher-Dose Neubotulinum Toxin Type A in Cervical Dystonia: A Multicenter Randomized Double-Blind Crossover Trial
Source: Toxins (Basel). 2026 Aug 28;18(9):370. doi: 10.3390/toxins18090370 (PMC13611609; doi:10.3390/toxins18090370)
Supplement: Supplementary file 1 [file toxins-18-00370-s001.zip › toxins-4532120-supplementary.pdf]

# Statistical Report V3 — FINAL

*Neu-BoNT-A 50 U versus 100 U cervical dystonia randomized two-period crossover trial*

This final V3 report uses the prespecified trial design as the analysis framework. Fifty participants were randomized 1:1: 25 to Neu-BoNT-A 100 U followed by 50 U and 25 to 50 U followed by 100 U. For statistical reporting, the two treatment phases are referred to as Period 1 and Period 2, consistent with the study design. Internal operational visit labels are not used in this report.

## 1. Analysis framework and conventions

- Randomized cohort: N=50; Sequence A, 100 U→50 U (n=25); Sequence B, 50 U→100 U (n=25).
- Treatment periods are designated Period 1 and Period 2, with crossover at Week 12.
- TWSTRS change is Week 12 minus pretreatment baseline; negative values indicate improvement.
- Treatment contrast is 100 U minus 50 U; negative values favor 100 U.
- No missing TWSTRS observations were imputed.
- The principal between-dose analysis is a linear mixed-effects crossover model with treatment, period, and randomized sequence as fixed effects and participant as a random effect.
- A Gaussian GEE with robust standard errors and an unstructured within-participant correlation is used as a robustness analysis because the fitted random-intercept variance is on/near the boundary.
- A first-period-only analysis is retained as a carryover-oriented sensitivity analysis.

## 2. Evaluable TWSTRS data

| Outcome | Period-level observations | Participants with ≥1 evaluable value | Complete pairs |
|---|---|---|---|
| TWSTRS total | 87 | 45 | 42 |
| Severity | 87 | 45 | 42 |
| Disability | 87 | 45 | 42 |
| Pain | 88 | 45 | 43 |

The randomized trial denominator remains N=50. The smaller numbers above reflect availability of calculable outcome changes for the relevant analysis, not a change in the randomized cohort. The principal crossover model uses all available evaluable period-level observations; complete-pair analyses are supportive sensitivity analyses.

## 3. Complete-pair TWSTRS sensitivity analysis

| Outcome | Paired N | Mean change 50 U | Mean change 100 U | 100 U−50 U | 95% CI | P | Cohen dz |
|---|---|---|---|---|---|---|---|
| TWSTRS total | 42 | +0.09 | −1.93 | −2.02 | −7.41 to +3.38 | 0.454 | −0.12 |
| Severity | 42 | −0.52 | −0.14 | +0.38 | −4.54 to +5.30 | 0.876 | +0.02 |
| Disability | 42 | +0.07 | −0.50 | −0.57 | −2.53 to +1.39 | 0.559 | −0.09 |
| Pain | 43 | +0.53 | −1.26 | −1.78 | −3.13 to −0.44 | 0.0104 | −0.41 |

Among participants with evaluable pain-change data under both regimens, the complete-pair pain analysis included 43 participants and favored 100 U. This analysis is supportive; the crossover model below is the principal reviewer-requested inference.

## 4. Principal crossover mixed-effects analysis

Model: change ~ treatment (100 U vs 50 U) + period (2 vs 1) + randomized sequence (100→50 vs 50→100) + participant random intercept, fitted by maximum likelihood.

| Outcome | Obs | Adjusted 100 U−50 U | 95% CI | P treatment | Period P | Sequence P |
|---|---|---|---|---|---|---|
| TWSTRS total | 87 | −1.76 | −5.99 to +2.47 | 0.414 | 0.594 | 0.936 |
| Severity | 87 | +0.58 | −3.05 to +4.21 | 0.753 | 0.659 | 0.549 |
| Disability | 87 | −0.49 | −2.12 to +1.14 | 0.554 | 0.283 | 0.483 |
| Pain | 88 | −1.83 | −2.93 to −0.73 | 0.0011 | 0.300 | 0.560 |

TWSTRS total, severity, and disability remained nonsignificant. TWSTRS pain favored 100 U after adjustment for period and randomized sequence (adjusted difference, −1.83 points; 95% CI, −2.93 to −0.73; P=0.0011). No statistically significant period or randomized-sequence effect was detected for any TWSTRS outcome.

## 5. GEE robustness analysis

The participant random-intercept variance was estimated on/near the boundary. A Gaussian GEE with robust standard errors and an unstructured within-participant working correlation was therefore used to assess robustness.

| Outcome | GEE 100 U−50 U | Robust 95% CI | Robust P |
|---|---|---|---|
| TWSTRS total | −1.69 | −6.93 to +3.55 | 0.527 |
| Severity | +0.66 | −4.23 to +5.56 | 0.791 |
| Disability | −0.48 | −2.16 to +1.20 | 0.573 |
| Pain | −1.83 | −3.04 to −0.62 | 0.0030 |

The pain finding remained statistically significant under the alternative covariance approach; the other TWSTRS outcomes remained nonsignificant.

## 6. First-period-only sensitivity and carryover interpretation

| Outcome | Period-1 N | 100 U−50 U | 95% CI | P |
|---|---|---|---|---|
| TWSTRS total | 44 | −1.94 | −7.61 to +3.73 | 0.494 |
| Severity | 44 | −0.53 | −5.68 to +4.63 | 0.838 |
| Disability | 44 | +0.09 | −1.86 to +2.05 | 0.925 |
| Pain | 44 | −1.50 | −3.18 to +0.17 | 0.077 |

Period 1 precedes exposure to the alternate randomized study dose and therefore avoids between-period carryover from that alternate dose, but it loses the within-participant crossover advantage. The pain estimate remained directionally concordant but less precise. A nonsignificant randomized-sequence term is not interpreted as proof of absence of biological carryover; residual biological carryover cannot be completely excluded.

## 7. Multiplicity sensitivity analysis

Holm's step-down procedure was applied across all 24 efficacy comparisons. For the four TWSTRS outcomes, the updated crossover-model P values were used; for the remaining secondary outcomes, the nominal P values reported in Table 2 were retained.

| Outcome | Nominal/updated P | Holm-adjusted P | Interpretation |
|---|---|---|---|
| TWSTRS total | 0.414 | 1.000 | Not significant |
| TWSTRS severity | 0.753 | 1.000 | Not significant |
| TWSTRS disability | 0.554 | 1.000 | Not significant |
| TWSTRS pain | 0.0011 | 0.025 | Significant |
| CDIP-58 psychosocial functioning | 0.001 | 0.024 | Significant |
| All other 19 efficacy comparisons | ≥0.060 | 1.000 | Not significant |

Both positive secondary findings remained significant after multiplicity adjustment: TWSTRS pain, Holm-adjusted P≈0.025, and CDIP-58 psychosocial functioning, Holm-adjusted P=0.024. No other efficacy comparison remained significant.

## 8. Integrated statistical interpretation

- The trial cohort remains N=50 with balanced 25:25 randomized sequence allocation.
- The overall TWSTRS total-score comparison is nonsignificant.
- TWSTRS pain consistently favors 100 U in the complete-pair, crossover mixed-effects, and robust GEE analyses.
- No significant period or randomized-sequence effect was detected.
- Residual biological carryover cannot be completely excluded.
- TWSTRS pain and CDIP-58 psychosocial functioning remain significant after Holm multiplicity adjustment.
- Pain remains a secondary endpoint, and no validated TWSTRS-pain-specific MCID anchor is available in the study materials.

## 9. Reviewer-facing wording for the crossover analysis

We thank the reviewer for this important recommendation. We verified the randomized treatment sequence according to the prespecified two-period crossover design (N=50; 25 participants assigned to 100 U followed by 50 U and 25 assigned to 50 U followed by 100 U) and reanalyzed the individual-level TWSTRS data using a linear mixed-effects crossover model including treatment, treatment period, and randomized sequence as fixed effects, with participant as a random effect. The adjusted between-dose difference remained nonsignificant for TWSTRS total (−1.76 points; 95% CI, −5.99 to 2.47; P=0.414), severity (+0.58; 95% CI, −3.05 to 4.21; P=0.753), and disability (−0.49; 95% CI, −2.12 to 1.14; P=0.554). TWSTRS pain favored 100 U (adjusted 100-U minus 50-U difference, −1.83 points; 95% CI, −2.93 to −0.73; P=0.0011). Neither period nor randomized sequence was statistically significant. Because the participant random-intercept variance was estimated at the boundary, we additionally performed a Gaussian GEE sensitivity analysis with robust standard errors and an unstructured within-participant

correlation; the pain result remained significant (−1.83 points; 95% CI, −3.04 to −0.62; P=0.003), whereas the other TWSTRS outcomes remained nonsignificant.

## 10. Manuscript-facing statistical wording

Methods — Between-dose TWSTRS comparisons were analyzed using a linear mixed-effects crossover model with treatment, period, and randomized treatment sequence as fixed effects and participant as a random effect. The randomized cohort comprised 50 participants, 25 in each sequence. Change was defined as Week 12 minus pretreatment baseline. Because the fitted random-intercept variance was at the boundary, a Gaussian GEE with robust standard errors and an unstructured within-participant correlation was used as a sensitivity analysis. A first-period-only analysis was additionally examined as a carryover-oriented sensitivity analysis. Holm's step-down procedure was applied across the 24 efficacy comparisons.

Results — In the crossover model, 100 U did not significantly differ from 50 U for TWSTRS total, severity, or disability. TWSTRS pain favored 100 U (adjusted difference, −1.83 points; 95% CI, −2.93 to −0.73; P=0.0011). Period and sequence effects were not significant. The pain result remained significant in the robust GEE analysis (−1.83 points; 95% CI, −3.04 to −0.62; P=0.003). TWSTRS pain and CDIP-58 psychosocial functioning remained significant after Holm adjustment (adjusted P≈0.025 and 0.024, respectively).
